# Dr. William B. Thomas

**Published:** 1907-04

**Authors:** 


Dr. William B. Thomas, of Ionia, Mich., died at his home March 5, 1907, aged 75 years. He graduated at the medical department, University of Buffalo, in 1857; he served during the civil war as surgeon of the 21st regiment, Michigan infantry volunteers; he was appointed United States marshal for the western district of Michigan in 1866, and five years afterward became superintendent of schools for Ionia County. He practised medicine about fifty years in Ionia, and became an invalid by reason of a fall received several years before his death.

